# Platypnea-Orthodeoxia Syndrome in Two Previously Healthy Adults: A Case-based Review

**DOI:** 10.4137/cmc.s2326

**Published:** 2009-04-09

**Authors:** Leon M. Ptaszek, Fidencio Saldana, Igor F. Palacios, Sean M. Wu

**Affiliations:** 1 Cardiology Division, Massachusetts General Hospital, Boston, MA 02114.; 2 Division of Cardiovascular Medicine, Brigham and Women’s Hospital, Boston, MA 02115.

**Keywords:** platypnea, orthodeoxia, interatrial shunting, dyspnea, patent foramen ovale, atral septal defect

## Abstract

We describe here the clinical manifestations of platypnea-orthodeoxia in two patients with interatrial shunting. In both cases, the patients were asymptomatic prior to developing additional cardiopulmonary issues that apparently enhanced right-to-left intracardiac shunting. The patients were both treated with percutaneously deployed occlusion devices, with excellent results. Symptoms and positional oxygen desaturation resolved after device placement in both cases. In addition, these patients remain symptom-free 30 months after device implantation.

## Introduction

Platypnea-orthodeoxia syndrome is defined by clinically observable dyspnea and decrease in arterial blood oxygen saturation that is most prominent when the patient is sitting or standing, and resolves when the patient is supine. This syndrome, first reported in 1949, may result from a number of cardiopulmonary processes, but is classically described as being the result of shunting of deoxygenated blood from the right atrium to the left atrium through an opening in the interatrial septum, most commonly a patent foramen ovale (PFO).^1^ Atrial septal defect (ASD) and atrial septal aneurysm (ASA) are less commonly implicated.^2^

The physiologic mechanism responsible for the positional nature of shunting is not fully understood. Under normal circumstances, left atrial pressure is 5–8 mmHg higher than right atrial pressure: this pressure difference is thought to prohibit right-to-left shunting through a PFO or small ASD, thus decreasing the likelihood of entrance of deoxygenated blood into the systemic circulation. Transient shunting of blood from the right atrium to the left may occur during specific circumstances, such as the Valsalva maneuver. Generally speaking, shunting sufficiently sustained to produce platypnea-orthodeoxia is not always present in patients with a PFO or a small ASD.^2^ This stands to reason, as platypneaorthodeoxia syndrome is quite rare, and PFO is thought to be present in as much as 25% o f the general population. Conditions that lead to an increase in right heart pressures can increase the right-to-left shunting and may lead to platypnea-orthodeoxia syndrome in previously asymptomatic people. A number of disease states have been shown to increase right atrial pressures above left atrial pressures, including: kyphoscoliosis, pulmonary vascular hypertension, and surgical lung resection.^3^,^4^ Pulmonary arteriovenous malformations (AVMs) have also been associated with increased right-to-left shunting with or without a PFO.^5^,^6^ Constrictive pericarditis and constrictive-effusive pericardial disease may also increase right heart pressures sufficiently to enhance right-to-left shunting through a pre-existing PFO.^7^ It is noteworthy that not all patients with persistent right-to-left shunting will have right heart pressures in excess of the normal range. Alteration of atrial geometry may also enhance the communication between the atria, potentially leading to right atrial remodeling and persistent shunting: such conditions include aneurismal expansion or elongation of the ascending aorta.^8^,^9^ Platypnea-orthodeoxia syndrome may also be caused by an intrapulmonary shunt, as is seen in the hepatopulmonary syndrome. In rare cases, primary pulmonary processes such as severe COPD, pulmonary embolus, or ARDS, can lead to a right-to-left shunt significant enough to lead to clinically evident platypnea-orthodeoxia.^2^

The diagnosis of platypnea-orthodeoxia syndrome is sometimes difficult to establish definitively at the bedside, as not all patients with interatrial shunting will present with clear postural changes in blood oxygen saturation. In some cases, the use of supplemental oxygen therapy may aid in the establishment of the diagnosis, as a right-to-left atrial shunt will prevent systemic oxygen saturation from reaching 100% even with additional inhaled oxygen. Frequently, imaging is required to make the diagnosis: echocardiography with Doppler mode and contrast injection is the most widely used modality for this purpose. Ideally, image procurement is performed with the patient in both the supine and upright positions.

We describe here the cases of two adults with platypnea-orthodeoxia syndrome who were treated at the Massachusetts General Hospital. In each case, the patient was asymptomatic and fully functional prior to an acute insult that led to a subacute increase in right heart pressures, which in turn led to an apparent increase in right-to-left shunting.

## Case #1

An 84-year-old woman with no previously reported cardiac or pulmonary disease was admitted due to progressive dyspnea over the course of four months. She had previously been very healthy, and had been engaged in daily physical labor until her symptoms prevented her from working. Her relatives reported that the patient had taken to bed, as her symptoms progressed to the point where even simple tasks around the home were exhausting. The patient and her relatives did not endorse any history of signs or symptoms that would raise concern for a respiratory illness: she reported only an intermittent, dry cough. There were no sick contacts or prior history of tuberculosis. The patient also did not endorse exertional chest discomfort or other symptoms that might suggest an anginal syndrome. She denied experiencing any fevers, but she did report cold insensitivity and brittle hair during this period.

On her initial evaluation, her blood oxygen saturation was noted to be low (96%) even with supplemental oxygen therapy (100% oxygen, delivered via face mask). Her other vital signs were normal. She was noted to be quite comfortable while supine, but quickly tired when she attempted to walk. Ambulatory oxygen monitoring revealed that her blood oxygen would drop to 86% within one minute after standing, even with face mask oxygen. Ambulation did not tend to lead to further drops in blood saturation. Similarly, rest while standing did not lead to recovery of her blood saturation: only return to a supine position led to recovery. Pulmonary auscultation revealed good air movement throughout both lung fields with clear breath sounds. Her neck veins were elevated to 10 cm H_2_O, but there was no other evidence of heart failure: no peripheral edema or clubbing was present. Cardiac auscultation revealed distant heart sounds with no extra heart sounds, rubs, or murmurs.

**Figure 1.**
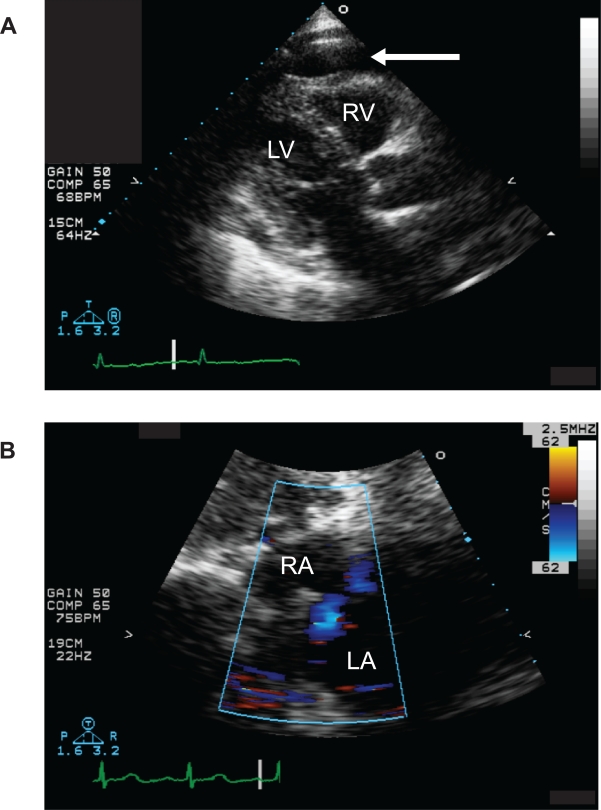
Panel **A**) TTE, parasternal long-axis view: a moderate, circumferential pericardial effusion is visible, with no evidence of pericardial mass or significant fibrin deposition (arrow). The silhouette of the right ventricular outflow tract, marked RV, reveals inversion of the free wall, indicating an increase in pericardial pressure. The left ventricle (marked LV) is also clearly visible in this view. Panel **B**) TTE, subcostal view with Doppler: the right atrium is marked RA and the left atrium is marked LA. The Doppler scan reveals a jet moving away (colored blue) from the probe, from the right to the left atrium.

Further evaluation included a chest x-ray, which revealed an enlarged cardiac silhouette but no abnormalities in the lung fields. ECG was within normal limits. Laboratory evaluation revealed no significant abnormality on Chemistry or complete blood count, but the thyroid stimulating hormone (TSH) level was markedly elevated at 46.5. Based on the presence of an enlarged cardiac silhouette on chest x-ray, a chest CT with contrast was performed. This study did not reveal any evidence of lung parenchymal abnormality or airspace disease. The pulmonary vasculature was also normal, with no evidence of pulmonary emboli, enlarged vessels, or arteriovenous malformations (AVMs). The cardiac structure did not appear grossly abnormal, but a moderate, circumferential pericardial effusion was noted. Based on this finding, a transthoracic echocardiogram (TTE) was performed. It is noteworthy that there was no clinical evidence of tamponade: the pulsus paradoxus was 5 mmHg. Left ventricular function was noted to be normal on the TTE, with no focal wall motion abnormalities. Both the right atrium (RA) and right ventricle (RV) were mildly compressed in early diastole, apparently due to the pressure from the pericardial fluid (Fig. 1A). No pericardial or extracardiac masses were found. Right ventricular systolic pressure was estimated to be in the high end of the normal range, based on the velocity of the tricuspid regurgitant jet. Doppler images of the interatrial septum revealed bidirectional shunting, with predominant right-to-left shunting through an apparent PFO/ASD (Fig. 1B). Images were obtained with the patient in the semirecumbent position: she did not tolerate image acquisition in the standing position.

Hypothyroidism was presumed to be the etiology of the pericardial effusion, as no other cause was evident. Based on the degree of right heart compression on TTF and clinical evidence of right-to-left interatrial shunting, it was thought that urgent treatment was indicated. As the pericardial effusion was thought to be the precipitant of the right-to-left shunting, the patient was brought to the cardiac catheterization lab for pericardiocentesis. The hope was that decompression of the pericardial sac would decrease right heart pressures and decrease the extent of right-to-left shunting. Right heart catheterization performed at the time of pericardiocentesis revealed elevated right atrial pressures (13 mmHg mean). The opening pressure of the pericardial sac was 15 mmHg. One-hundred seventy ml of straw-colored fluid was collected: right atrial pressure was reduced to 7 mmHg and pericardial pressure was reduced to 0 mmHg. Further analysis of this fluid revealed that it was transudative, with no evidence of infection or malignancy.

After drainage of the pericardial fluid, the patient was improved, but not back to her prior baseline. Her oxygen saturation while supine was 98% with 100% O_2_ delivered via face mask. In the standing position, her oxygen saturation dropped to 90%. A repeat TTE revealed a bidirectional shunt, with predominant right-to-left shunt with no change in her estimated right ventricular systolic pressure. The patient remained symptomatic with standing and ambulation, and was comfortable only when supine. Therefore, it was determined that her PFO would need to be closed in order to resolve her shunt.

**Figure 2.**
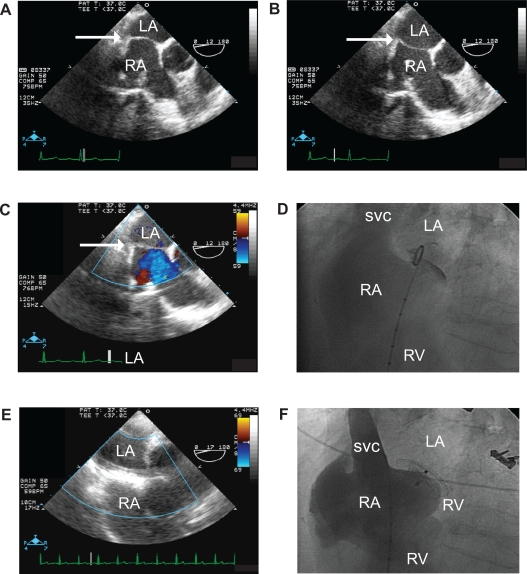
Panels **A** and **B**) Intra-procedural TEE, prior to device implant. The left atrium is visible at the top of the image (marked LA), with the right atrium directly beneath (marked RA). The interatrial septum is marked with a white arrow in both images. Comparison of panels A and B reveals the mobility of the interatrial septum. Panel **C**) Intra-procedural TEE with Doppler, prior to device implant. The left atrium is visible at the top of the image (marked LA). The interatrial septum is marked with an arrow. Doppler imaging reveals a flow jet emanating from the interatrial septum: this blue-colored jet is moving away from the probe, indicating a left-to-right shunt is present while the patient is in the supine position. Panel **D**) Intra-procedural angiogram (LAO cranial view) prior to device implant. The right atrium is visible on the left side of the image, opacified with contrast (marked RA). The superior vena cava (marked SVC) is also marked with contrast. The right ventricle, marked RV, contains a small amount of contrast. A pigtail catheter is present in the right atrium, with the tip of the catheter in the PFO flap. A small wisp of contrast is visible passing through the PFO into the left atrium (marked LA). Panel **E**) Intra-procedural TEE with Doppler, after device implant. As in the other TEE panels, the left atrium (marked LA) is visible at the top of the image, with the right atrium directly beneath (marked RA). The echo-dense structure in the area of the interatrial septum is the occlusion device. There is no significant Doppler flow across the device. Panel **F**) Intra-procedural angiogram (LAO cranial view, identical to the view in 2D) after the placement of the occlusion device. The pigtail catheter present in the right atrium. (RA) was used to opacify the right atrium, superior vena cava (SVC), and right ventricle (RV) with contrast. The occlusion device is visible at the boundary between the left and right atria. No passage of contrast into the left atrium (LA) is visible.

The patient was brought back to the cardiac catheterization lab, for occlusion of her PFO with a percutaneously deployed (Amplatzer) occlusion device. Intra-procedural transesophageal echocardiography (TEE) and angiography revealed a mobile interatrial septum (Figs. 2A, 2B). As intraprocedural TEE could only be performed in the supine position, only the left-to-right component of the apparently bidirectional shunt could be visualized (Fig. 2C). An intraprocedural angiogram, performed prior to device implantation, revealed an open channel between the atria (Fig. 2D). Post-deployment TEE and angiography verified that the device was well-seated and shunting had been stopped (Figs. 2E, 2F). Post-procedure saturation testing revealed that her resting arterial oxygen saturation had increased to 100% in both the supine and standing positions with no need for supplemental oxygen. Most importantly, after physical therapy, the patient returned to her prior activity baseline. The patient has remained asymptomatic 30 months after device implantation. Serial TTEs have revealed no further right-to-left shunting and no recrudescence of her pericardial effusion with thyroid replacement therapy.

## Case #2

A 29-year-old woman with no prior cardiac history sought medical attention due to progressive dyspnea over the course of six months. Her prior medical history was significant for an episode of idiopathic rhomboencephalitis two years prior, complicated by a persistent neuropathy. Since that time, she had been treated with carbamazepine and had experienced no additional neuropathic problems, but she gained in excess of 100 pounds. As a result of this weight gain, the patient developed obstructive sleep apnea (OSA) approximately fourteen months after her rhomboencephalitis. This was treated successfully with nighttime CPAP. A pulmonary evaluation at that time did not reveal any evidence of COPD. Detailed questioning at that time did not reveal any risk factors for pulmonary disease. Several months after the initiation of CPAP, the patient reported dyspnea and a decline in her exertional capacity despite adherence with therapy.

Due to her dyspnea, she initially sought care at an urgent care facility close to her home: there, she was found to be hypoxemic, with arterial oxygen saturations as low as 87%. As a result, she was transferred to our hospital. At the time of her initial evaluation, she was noted to be resting comfortably in a supine position, with oxygen saturation 98% on 2 liters/minute nasal cannula supplementation. All other vital signs were within normal limits, and her pulsus paradoxus was not elevated. The patient was morbidly obese, but there were no other noteworthy findings on physical exam. Of note, her cardiopulmonary exam was within normal limits, and there was no other clinical evidence of fluid overload: her jugular venous pulse was at 6 cm H_2_O, and no lower extremity edema was present. There was no evidence of clubbing of the fingers. Attempts at ambulation revealed that the patient was not able to stand without difficulty: she would become dyspneic, with a drop in her oxygen saturations to 87% on 2 liters/minute nasal cannula oxygen. She could only walk a few steps, and noted that her symptoms reached maximum intensity just after standing. As with the patient described in Case #1, only rest in a supine position, not a standing position, led to relief.

**Figure 3.**
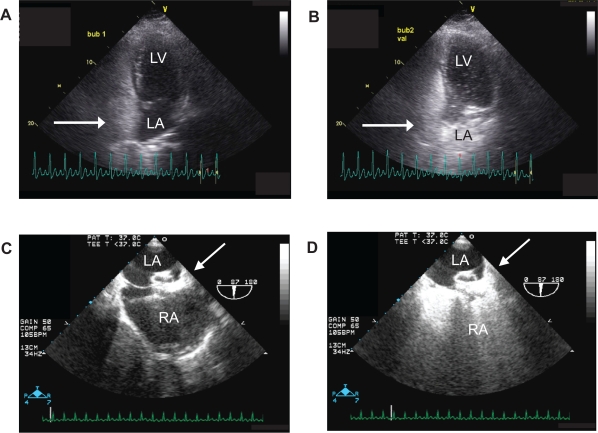
Panel **A**) TTE, apical four-chamber view. This image was obtained while the patient was breathing normally. Bubble contrast has opacified the right atrium (arrow). There is no evidence of bubble contrast in the left heart: left atrium and left ventricle are marked LA and LV, respectively. Prolonged imaging after contrast injection did not reveal late appearance of contrast in the left heart, indicating that a pulmonary shunt is not likely present (data not shown). Panel **B**) TTE, apical four-chamber view. This image was obtained during the Valsalva maneuver. Bubble contrast has opacified the right heart (arrow). Contrast has opacified the left atrium (LA) and a significant amount of contrast is visible in the left ventricle (LV): this contrast appeared within three cardiac cycles after contrast reached the right heart. Panel **C**) Intraprocedural TEE without bubble contrast, performed after the occlusion device was percutaneously deployed. The left atrium (LA) is visible at the top of the image, with the interatrial septum and the right atrium (RA) beneath. The echo-dense mass in the interatrial septum is the device (arrow). Panel **D**) Intraprocedural TEE with bubble contrast, performed after the occlusion device was percutaneously deployed. The view in this image is identical to that in Figure 3C with the exception of contrast. Opacification of the right atrium (RA) is visible in this image, with no evidence of passage of contrast across the device (arrow) into the left atrium (LA).

Chest x-ray and ECG did not reveal any concerning abnormalities. Similarly, laboratory evaluation was essentially normal. Chest CT with intravenous contrast did not reveal any evidence of significant pulmonary parenchymal or airspace disease. Her pulmonary vasculature was also normal, with no evidence of thromboembolic disease or AVMs. As her platypnea and orthodeoxia raised suspicion for interatrial shunting, an echocardiogram was performed. This study did not reveal any obvious structural abnormalities, but was limited by her body habitus. Echocardiography was attempted in the standing position, but was not successful as the patient was too symptomatic in this position to allow for good image acquisition. Attempts at estimation of right ventricular systolic pressure were not successful. Doppler imaging of the interatrial septum did not reveal unequivocal evidence of PFO/ASD or shunting. Bubble study, however, did reveal a right-to-left shunt. This shunt was not present at all times: a Valsalva maneuver was required to elicit shunt of contrast from the right to the left atrium (Figs. 3A, 3B).

Since her presenting symptoms were severe, and were thought to be caused by right-to-left interatrial shunting, she was considered for device closure of her PFO/ASD. She was brought to the cardiac catheterization laboratory, where a right-heart catheterization was performed. This revealed elevated right heart pressures: RA 15 mmHg, RV 36/15 mmHg. Pulmonary capillary wedge pressure was within normal limits: the elevation in her right heart pressures was presumed to be secondary to her body habitus-related sleep apnea.

Trans-esophageal echocardiography was performed during cardiac catheterization. This verified the presence of a small aperture at the inferior aspect of the interatrial septum. As with the patient described in Case #1, a septal occlusion device (Amplatzer) was used successfully to close the aperture. Post-deployment use of bubble contrast revealed that the device was well-seated and that no residual right to left shunting was present (Figs. 3C, 3D). After deployment of the aforementioned device, the patient’s oxygen saturations in the supine and standing positions were re-checked, and were found to be 100% in both positions with no supplemental oxygen. The patient also reported disappearance of her presenting complaint. Soon thereafter, her exertional capacity returned to her pre-illness baseline. She has remained symptom-free for 30 months after device placement.

## Discussion

Platypnea-orthodeoxia syndrome is a relatively rare manifestation of shunting of blood from the right to the left heart, generally through a defect in the interatrial septum. PFOs are highly prevalent in the general population and are cited as the most common underlying cause of this syndrome. Given the large difference between the prevalence of PFO and platypnea-orthodeoxia syndrome, it appears that this interatrial defect is itself not sufficient to produce clinically evidence issues in most cases. It is suspected that platypnea-orthodeoxia syndrome is present in patients with an existing PFO in whom there is an additional disease process that increases right heart pressures. There are several examples of such processes, including pulmonary hypertension and pericardial effusion. The platypneaorthodeoxia noted in the patient described in Case #1 appears to have been precipitated by her hypothyroidism-related pericardial effusion. In the patient described in Case #2, the clinical syndrome appears to have been set off by pulmonary hypertension related to her OSA. Absent these additional processes, the PFO would likely have remained clinically quiescent. Transient hemodynamic changes, such as those associated with the Valsalva maneuver, may be sufficient to lead to the passage of paradoxical emboli, but do not appear to be sufficient to produce platypnea-orthodeoxia syndrome. In many cases of platypnea-orthodeoxia syndrome, the positional shifts on arterial oxygen saturation levels are not as dramatic as those associated with the cases discussed here. This emphasizes the point that right-to-left shunting ought to be actively considered in patients whose blood oxygenation falls short of a predicted level (with oxygen supplementation) even when a clear positional component is not present. Correction of the non-cardiac insult (e.g. pulmonary hypertension) may be sufficient to improve, if not correct, the relative hypoxia associated with the shunt. In many cases, however, correction of the interatrial shunt through physical occlusion may not be avoidable. In Case #1, drainage of the pericardial effusion led to a decrease in right heart pressures and an improvement, but no a disappearance in her clinical syndrome. In Case #2, the patient was already receiving appropriate therapy for OSA when her platypnea-orthodeoxia-related symptoms started.

Surgical correction is traditionally the treatment of choice for platypnea-orthodeoxia associated with interatrial shunting. For patients with suitable anatomy, such as PFO or ASD sufficiently separated from adjoining structures, closure with a percutaneously deployed device represents a less invasive alternative.^10^ Several registries have documented the long-term safety and efficacy of the percutaneous approach.^11^–^13^
